# Contrast Sensitivity of Thyroid Associated Ophthalmopathy Patients without Obvious Optic Neuropathy

**DOI:** 10.1155/2013/943789

**Published:** 2013-12-24

**Authors:** Ümit Beden, Sümeyra Kaya, Volkan Yeter, Dilek Erkan

**Affiliations:** ^1^Department of Ophthalmology, Memorial Hospital, Istanbul, Turkey; ^2^Department of Ophthalmology, Ahi Evran University, Kırşehir, Turkey; ^3^Department of Ophthalmology, Erzincan University, Mengücek Gazi Training and Research Hospital, Göz Hastalıkları, 24000 Erzincan, Turkey; ^4^Department of Ophthalmology, Dünya Göz Hospital, Samsun, Turkey

## Abstract

*Purpose*. To compare the contrast sensitivity levels of thyroid associated ophthalmopathy (TAO) patients without obvious optic neuropathy with those of healthy people. *Methods*. Forty eyes of 20 TAO patients without dysthyroid optic neuropathy and 40 eyes of 20 healthy subjects were evaluated in this prospective case-controlled study. The contrast sensitivity functions (CSFs) of all subjects were measured by the functional acuity contrast test (FACT) in five frequencies which were 1,5 cpd (A), 3 cpd (B), 6 cpd (C), 12 cpd (D), and 18 cpd (E). Results were compared for both groups, and a correlation of CSF with Hertel and clinical activity scores was assessed. *Results*. There was no statistically significant difference between TAO patients and control groups for age and sex. TAO patients had lower levels than the control group in all the frequencies of CSFs (*P* < 0.05) and the difference in contrast sensitivity functions between the groups seems to be more significant in higher frequencies (B, C, D, and E) (*P* < 0.001). *Conclusions*. TAO patients without DON can have contrast sensitivity loss and this would probably imply subtle optic nerve dysfunction in early disease phase.

## 1. Introduction

Thyroid associated ophthalmopathy (TAO) is related to autoimmune inflammation of orbital tissues [1]. Although the effects of the disease on lids, cornea, extraocular muscles, and ocular soft tissues can be defined and classified, the diagnostic criteria of dysthyroid optic neuropathy (DON) are still up to debate. In spite of different tests used (visual acuity, visual field, color vision, contrast sensitivity, pupillary light reflexes, and VEP), diagnosis in mild neuropathy is usually subjective unlike this in severe cases [2–4]. Thereby, the stress over the optic nerve must be evaluated by the qualitative methods and the decision of treatment should be supported by objective criteria.

Although determination of visual acuity level is the most common and easy way for the evaluation of visual system, visual acuity may not be affected in some TAO patients with optic nerve under stress [3, 5]. Contrast sensitivity function (CSF) has been shown to be sensitive for the detection of visual disturbance in many ophthalmic disorders before the impairment of visual acuity and it is widely used in diagnosing and following these diseases, such as glaucoma [6], multiple sclerosis [7, 8], optic neuropathies [9, 10], and also Grave's ophthalmopathy [11]. Although we need a threshold for therapeutic intervention, we think that functional disturbance in optic nerve probably starts with early stages of TAO.

In this study, we assessed contrast sensitivities of the subjects with thyroid associated ophthalmopathy, who have no obvious optic neuropathy compared to those of healthy subjects with similar demographic properties.

## 2. Methods

### 2.1. TAO and Control Groups

In this study, 20 patients followed up for TAO in the Clinics of Ophthalmology Department, Ondokuz Mayıs University, Samsun, Turkey, and 20 healthy subjects were included. A full ophthalmologic examination for best corrected Snellen visual acuity, corneal and conjunctival appearance, pupillary light reflexes and presence of a RAPD, degree of lid retraction, exophthalmometer reading, intraocular pressure obtained by Goldmann applanation tonometry, Ishihara color plate performance, retina and optic disc appearance by 90 D lens, and cataract/lens density was carried out for all subjects. Schirmer test was not performed for our patients, because none of our patients had any symptoms and signs for dry eye, and the sensitivity of this test for detection of the dry eye is also controversial.

Ophthalmic signs of TAO patients were graded by NOSPECS classification [12] (N: no signs and symptoms. O: only signs, limited to upper lid retraction and stare, with or without lid lag. S: soft tissue involvement, edema of conjunctiva and lids, conjunctival injection, and so on. P: proptosis. E: extraocular muscle involvement. C: corneal involvement due to lagophthalmos. S: sight loss due to optic neuropathy). In the diagnosis of TAO, history and clinical signs, thyroid function tests, thyroid related autoantibody levels, and presence of fusiform enlargement of the extraocular muscles in the axial sections of orbital CT were investigated. Clinical activity score was calculated for every patient as previously reported [13].

Twenty healthy subjects without any systemic or ocular pathology—except minor refractive errors—or past ocular surgery whose visual acuities were 20/20 with or without refractive correction were included as control group in the study. The subjects, who had significant impairment in visual acuity or additional nonrelated conditions (such as glaucoma, amblyopia, cataract/lens opacity, dry eye, mild macular or retinal disease, etc.) which may affect contrast sensitivity function, were excluded from the study. Any degree of corneal involvement (dry eye, punctuate epithelial defect, corneal ulcer, or keratitis) was accepted as exclusion criterion. The study was approved by the Human Research Ethics Committee, University of Ondokuz Mayıs, Samsun, Turkey. It was conducted in accordance with the tenets of the Declaration of Helsinki and we obtained verbal assent from all subjects.

### 2.2. Contrast Sensitivity Function Tested by FACT

The contrast sensitivity of both groups was measured with the Functional Acuity Contrast Test (FACT; Stereo Optical Co. Inc., Chicago, IL, USA). This allows presentation of sine-wave gratings of different spatial frequencies: 1.5 (A), 3 (B), 6 (C), 12 (D), and 18 (E) cycles per degree (cpd). During testing, the distance between the patient and the chart of FACT was 3 m and the chart was kept in same height with the patient. The measurements were taken at the photopic level and under stable illumination conditions (150 cd/m^2^). Patients adapted to the illumination level for 5 minutes before testing. The test was performed by asking patients the direction of the gratings, respectively, from the first line to down and from left column to right. The responses of the patient were recorded to the contrast sensitivity form of FACT for all frequencies (A, B, C, D, and E). Both eyes of the subjects were separately measured by covering the fellow eye. Forty eyes of TAO group and 40 eyes of control group were measured for contrast sensitivity function by this method.

### 2.3. Statistical Analysis

The data about age and sex of the patients, intraocular pressure, visual acuity, and spherical equivalent of the eyes and the contrast sensitivity results taken by FACT were recorded and statistical analysis was performed by SPSS 15 for Windows (SPSS Inc., Chicago, IL, USA). Kruskal-Wallis test and Mann-Whitney *U* test were used for comparing the values of the groups. Countable variables were compared by Chi-square test between the groups. For evaluation of the correlation of CSF between the measurements of Hertel exophthalmometer and clinical activity score, Spearman's correlation test was performed. For the statistical tests, a *P* value of less than 0.05 indicated statistical significance.

## 3. Results

Thirty-five percent of TAO patients (*n* = 7) were males and 65% of them were females (*n* = 13). In control group, 45% of them (*n* = 9) were males and the rest of them (55%, *n* = 11) were females. There was no statistically significant difference between the groups for gender (*P* > 0.05, *χ* ^2^ = 0.104; Chi-square test). The mean ages in TAO and control groups were 43.22 ± 9.08 years (range: 21–60 years) and 43.0 ± 9.88 years (range: 20–60 years), respectively. There was no significant difference between the groups for age, refractive error, intraocular pressure (IOP), and color vision level. The comparison of the groups for age, gender, spherical equivalent, IOP, and color vision level is shown in Table 1.

The TAO patients were evaluated by thyroid function tests and it was found that one patient had hypothyroidism, one of them was in euthyroid stage, and the rest of them (*n* = 18) had hyperthyroidism. The mean time of thyroid disease was 35.55 ± 40.6 months (range: 1–120 months). Hyperthyroidism is most common in early phase of TAO. Because the patients in early phase with no DON are the target population for our study, hyperthyroidism is the most common endocrinological status in our study as a natural consequence.

The mean values of visual acuity level in TAO and control groups were 0.98 ± 0.48 and 1.0 ± 0.00 (acuity levels in decimal). There was statistically significant difference for visual acuity level between the groups. The visual acuity of a patient was 0.8/1.0 and three of them had 0.9/1.0 of visual acuity levels in TAO group.

**Figure 1:**
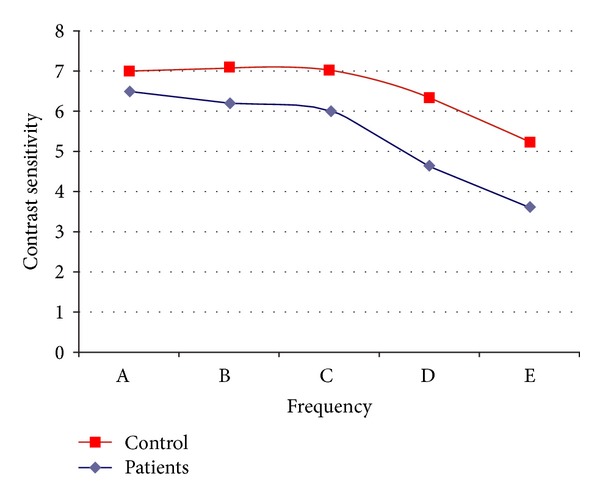
Decreased contrast sensitivity for all frequencies in TAO patients was shown in the graph.

When the CSFs of the patients in two groups were compared in five different spatial frequencies, it was found that TAO patients had contrast sensitivity level significantly lower than control group in all frequencies (*P* < 0.05 for all frequencies) (Table 2). When four patients, whose visual acuity was lower than 1.0/1.0, were excluded from the TAO group, the statistical difference between the contrast sensitivity levels of the groups has not changed (Table 3). Contrast sensitivity of patients and control in 5 different spatial frequencies are graphically compared in Figure 1.

In TAO group, the mean measurement of Hertel exophthalmometer was 19.88 ± 2.71 mm and the mean of clinical activity score was 0.60 ± 0.81. The contrast sensitivity levels were not correlated with exophthalmos or clinical activity scores in TAO group (Table 4).

When patients were classified according to NOSPECS classification, 6 eyes were in “N” stage, 26 eyes were in “O” stage, 6 eyes were in “S” stage, 16 eyes were in “P” stage (11 mild, 5 moderate), 3 eyes were in “E” stage, and 8 eyes were in “C” stage. An eye can be classified into two or more different NOSPECS stages at the same time. No patient had DON. While there was statistically significant difference in contrast sensitivity level between TAO patients and control group, contrast sensitivity level was not significantly different between all different subgroups of NOSPECS in TAO group (Table 5).

## 4. Discussion

Thyroid associated ophthalmopathy (TAO) is characterized by edema and infiltration of the extraocular muscles and orbital tissue and increased muscle volume may cause pressure over the optic nerve at the orbital apex resulting in visual impairment [2–5, 14, 15]. Unless the optic nerve function decreases under the threshold level (usually accepted as 20/30), optic nerve stress may not be detected. The signs and symptoms of DON are decreased visual acuity, presence of relative afferent pupillary defect, impairment of color vision, and abnormal visual evoked potential. The decrease of visual acuity may not be dramatic and, in half of the dysthyroid optic neuropathy patients, visual acuity level is 20/40 and over [3]. Defects on visual field test and color vision can be detected in only 5% of the patients [3, 5, 16]. DON is generally diagnosed after irreversible visual loss has occurred. At subclinical stage, the detection of early external congestive symptoms and active disease signs may be important for early treatment [17]. Noninvasive and sensitive diagnostic tests are needed for the detection of DON before the development of irreversible visual deficit. CSF test is noninvasive and sensitive test for the determination of early effects of glaucoma [6], multiple sclerosis [7, 8], optic neuropathies, and compressive lesions of anterior visual pathway [9, 10] and it also enables detecting the central deficits of visual field that may be missed by Snellen vision test [18]. In our prospective study, we evaluated the function of optic nerve by contrast sensitivity function test and we found that it is decreased in all TAO patients. It was thought that optic nerve functions may be affected in early stages of TAO before clinically evident dysthyroid optic neuropathy. Suttorp-Schulten et al. examined 38 eyes with DON and 34 eyes with only TAO by contrast sensitivity function test and they compared the contrast sensitivity results of these TAO patients with or without DON and those of 74 healthy subjects [19]. They found that the CSFs of the patients with DON significantly decreased and the TAO patients without DON also had lower contrast sensitivity level than the healthy subjects. The sensitivity loss of the patients with DON was higher at lower frequencies (1–5 c/deg) than that of the patients without DON [19]. So, they reported that CSF test may be beneficial for the discrimination of the TAO patients complicated with DON from those without DON [19]. Mourits et al. evaluated the diagnostic methods and spatial vision and measured the CSFs in 34 eyes of 19 DON patients [20]. They found that the CSF may be deteriorated even in the patients who have relative conserved visual acuity and they observed that CSF was improved after orbital decompression surgery in 33 eyes [20].

Grave's ophthalmopathy has several different ophthalmic manifestations that may potentially induce some abnormalities in CSF. One of them is secondary glaucoma. Five percent of the patients who suffered from endocrine exophthalmos have secondary glaucoma due to increased orbital venous pressure [21]. Several studies have shown that the patients with primary open angle glaucoma have sensitivity loss in temporally modulating gratings and in low and moderate spatial frequencies of contrast [22–26]. It was known that the patients with increased intraocular pressure (IOP) and with no visual field defect may have decreased CSF in low and moderate spatial frequencies [24, 26, 27]. As TAO patients with increased IOP may have deteriorated CSF, in some patients it can mask the compressive damage at the orbital apex. de Marco et al. compared the CSFs of 20 eyes with uncomplicated TAO, those of 14 eyes with increased IOP more than 24 mmHg, and those of 12 eyes with DON and CSFs of 40 healthy eyes [18]. They reported that the patients with uncomplicated TAO had normal CSF in contrast to the results of Suttorp-Schulten's study [19] and the eyes with DON had contrast sensitivity disruption at overall intermediate-high spatial frequencies [18]. When eyes with DON were compared, those with increased IOP or suspected glaucoma had pronounced sensitivity loss in the low frequencies (0.18–0.70 c/deg). They showed that CSF can detect visual function impairment noninvasively in patients with complicated endocrine ophthalmopathy and it can distinguish the eyes with ocular hypertension or suspected glaucoma and eyes with DON when a temporal modulation of 6.87 Hz and gratings of low spatial frequency (<1 c/deg) are used [18]. The difference of the results between Suttorp-Schulten's and Rocco's study for CSF in uncomplicated TAO patients may occurr because in the study of Suttorp-Schulten et al. [19] the patients were not classified for secondary glaucoma and the effect of the elevated intraocular pressure over the CSFs was not known. In our study, all TAO had no secondary glaucoma or elevated IOP; the mean IOP of TAO patients and control group was 14.82 mmHg and 14.77 mmHg, respectively. There was no statistically significant difference between these groups for IOP. Thereby, the effect of elevated IOP or secondary glaucoma over the CSFs was eliminated in our study.

In this prospective study, we found statistically significant decrease of CSF in TAO patients for all frequencies when compared with healthy subjects. None of the patients in TAO group had any sign of DON or afferent papillary defect and all patients had nearly normal visual acuity and normal color vision. Thereby, our study shows that optic nerve functions may be affected in early stages of thyroid associated ophthalmopathy. The reason for this effect on CSF in early stage is not exactly known. There was no significant correlation between Hertel measurements, clinical activity scores, and CSFs in TAO patients. When the groups classified by NOSPECS were compared to each other for CSF, we found no difference between the NOSPECS groups.

We used Ishihara color plates for the evaluation of color vision in our study because it is a routine clinical test used for detecting whether optic neuropathy exists or not in a patient. If the color tests more sensitive than Ishihara had been used in our study, it would most probably support our study results and these tests could probably detect the impairment of optic nerve functions in early phase of the disease as contrast sensitivity function test. For this reason, further multicentered studies with larger sample which are supported by various clinical sensitive tests are needed.

Optic nerve stress in early stage of TAO may change the thoughts and the aspects of the clinicians for the disease. In active stage of TAO, the signs of inflammation are pronounced and the inflammation regresses in inactive stage. Determination of the disease stage is important for decision of the treatment. Immunosuppressive treatment is indicated for TAO patients with active inflammation or DON. Decompression surgery on the other hand is usually reserved for inactive patients and those with DON. In these settings most of the patients are followed up on a wait-and-see policy unless severe inflammation and DON develop. Hence, early presentation of optic nerve stress may change the treatment protocols of the disease and early diagnosis and treatment may alter the course and progression of the disease and may lead to obtaining better visual and cosmetic outcome.

Although this study had small sample size, it was found that the patients with TAO with no DON can have contrast sensitivity loss and this would probably imply subtle optic nerve dysfunction in early disease phase.

## Figures and Tables

**Table 1:** Comparison of age, gender, IOP, refractive error, and color vision between TAO and control group.

	TAO	Control	*P *
Age			
(mean ± SD)	43.2 ± 9.08	43.0 ± 9.88	.74
(min–max)	21–60	20–60	
Gender			
Female	13 (65%)	11 (55%)	.74
Male	7 (35%)	9 (45%)	
IOP (mmHg)			
(mean ± SD)	14.82 ± 2.50	14.77 ± 2.36	.86
(min–max)	(10–18)	(10–18)	
Spherical equivalent (Dioptre)			
(mean ± SD)	0.70 ± 1.41	0.72 ± 0.93	.21
(min–max)	(0.0–8.0)	(0.0–4.50)	
Color vision	9.0 ± 1.01	9.3 ± 0.96	.43

TAO: thyroid associated ophthalmopathy.

IOP: intraocular pressure.

SD: standard deviation.

**Table 2:** Comparison of contrast sensitivity functions between TAO patients and controls in 5 different spatial frequencies.

Frequency	TAO patients Mean ± SD (min–max)	Controls Mean ± SD (min–max)	*P *
A	6.47 ± 1.08 (5–8)	6.97 ± 0.73 (6–8)	.039
B	6.17 ± 0.95 (4–8)	7.07 ± 0.61 (6–8)	<.001
C	6.00 ± 1.08 (2–8)	7.00 ± 0.64 (6–8)	<.001
D	4.65 ± 1.42 (1–7)	6.32 ± 0.85 (5–8)	<.001
E	3.62 ± 1.79 (1–7)	5.22 ± 1.56 (2–8)	<.001

TAO: thyroid associated ophthalmopathy.

SD: standard deviation.

Letters indicate different spatial frequencies in FACT: 1.5 (A), 3 (B), 6 (C), 12 (D), and 18 (E) cycles per degree.

**Table 3:** *P* values of the difference in contrast acuity between TAO patients and healthy subjects for 5 different spatial frequencies in subjects with 1.0/1.0 visual acuity.

Frequency	A	B	C	D	E

*P* value	.043	<.001	<.001	<.001	.001

TAO: thyroid associated ophthalmopathy.

Letters indicate different spatial frequencies in FACT: 1.5 (A), 3 (B), 6 (C), 12 (D), and 18 (E) cycles per degree.

**Table 4:** Significance of correlation between contrast sensitivity levels and Hertel exophthalmometer measurements and clinical activity score (CAS) in patients with TAO.

	Mean ± SD (min–max)	*P* values				
		A	B	C	D	E
Hertel Ex. (mm)	19.88 ± 2.71 (16–25.5)	.559	.364	.561	.910	.471
CAS	0.60 ± 0.81 (0.0–3.0)	.952	.826	.648	.690	.222

Hertel Ex.: Hertel exophthalmometer measurement. It is used for the measurement of exophthalmos level.

CAS: clinical activity score.

SD: standard deviation.

Letters indicate different spatial frequencies in FACT: 1.5 (A), 3 (B), 6 (C), 12 (D), and 18 (E) cycles per degree.

**Table 5:** *P* values of differences in contrast acuity between all different subgroups of NOSPECS in TAO group.

Groups	Frequency				
	A	B	C	D	E
O	.181	.727	.684	.695	.446
S	.377	.984	.212	.969	.908
P	.338	.593	.546	.157	.250
E	.055	.885	.923	.674	.962
C	.916	.575	.640	.213	.959

TAO: thyroid associated ophthalmopathy.

NOSPECS classification was described in Section 2.

Letters indicate different spatial frequencies in FACT: 1.5 (A), 3 (B), 6 (C), 12 (D), and 18 (E) cycles per degree.
